# Dietary magnesium and risk of cardiovascular and all-cause mortality after myocardial infarction: A prospective analysis in the Alpha Omega Cohort

**DOI:** 10.3389/fcvm.2022.936772

**Published:** 2022-08-12

**Authors:** Ilse Evers, Esther Cruijsen, Iris Kornaat, Renate M. Winkels, Maria C. Busstra, Johanne M. Geleijnse

**Affiliations:** ^1^Division of Human Nutrition and Health, Wageningen University and Research, Wageningen, Netherlands; ^2^Nutrition & Healtcare Alliance, Ede, Netherlands

**Keywords:** dietary magnesium, myocardial infarction, cardiovascular disease, mortality, patients

## Abstract

**Background:**

An adequate intake of magnesium has been associated with lower risks of cardiovascular disease (CVD) and all-cause mortality in population-based studies. Whether an adequate magnesium intake is important for reducing long-term mortality risk after myocardial infarction (MI) is not yet clear.

**Objective:**

We examined magnesium intake in relation to CVD, all-cause and coronary heart disease (CHD) mortality, on top of drug treatment, in patients who had experienced an MI.

**Methods:**

We included 4,365 Dutch patients aged 60–80 y from the Alpha Omega Cohort with a history of MI <10 y before study enrollment. Dietary data over the past month were collected at baseline using a 203-item validated food frequency questionnaire from which magnesium intake was calculated. Patients were followed for cause-specific mortality through December 2018. HRs for mortality in tertiles of energy adjusted magnesium intake were obtained from multivariable Cox proportional hazard models, adjusting for age, sex, education, obesity and other lifestyle and dietary factors. Associations were also studied in relevant subgroups, including patients with diabetes and diuretics users. Restricted cubic splines were used for studying the continuous association of magnesium intake with CVD mortality.

**Results:**

The average magnesium intake was 302 ± 78 mg/day and 28% of male and 33% of female patients had adequate intakes. Magnesium containing supplements were used by 5.4% of the cohort. During a median follow-up of 12.4 years (48,473 person-years), 2,035 patients died, of which 903 from CVD and 558 from CHD. Higher magnesium intakes (>320 g/d), compared to the reference group (<283 mg/d), were associated with a lower risk of CVD mortality (HR: 0.72; 95% CI: 0.54–0.98) and all-cause mortality (HR: 0.78; 95% CI: 0.64–0.95) in the fully adjusted model. A non-significant inverse association was found for CHD mortality. Associations for CVD mortality were slightly stronger in diuretic users (HR: 0.55; 95% CI: 0.34–0.89). Results were similar after excluding magnesium supplement users.

**Conclusion:**

An adequate intake of magnesium may be important for lowering long-term mortality risk after MI, especially in patients treated with diuretics. The Alpha Omega Trial was registered at clinicaltrials.gov as NCT03192410.

## Introduction

Population-based studies have shown inverse associations of dietary magnesium with risk of cardiovascular diseases (CVD) and risk of hypertension, metabolic syndrome and type 2 diabetes (T2D) (1–4). Bagheri et al. showed a relative risk for CVD mortality of 0.92 (95% CI: 0.87–0.97) for high vs. low dietary magnesium intake (range from 126 to 523 mg/d) after pooling of 17 studies in which adjustment was made for total energy intake (5). In a meta-analysis of 11 prospective cohort studies by Del Gobbo et al. (3), dietary magnesium (per 200 mg/d increment) was associated with a 22% lower risk (RR: 0.78; 95% CI: 0.67–0.92) of coronary heart disease (CHD) events. Magnesium plays an essential role in blood glucose control, blood pressure regulation and myocardial metabolism (6, 7). Magnesium is derived from fiber-rich foods such as whole grains, green vegetables, legumes and nuts, and also from dairy (6). Dietary reference values (adequate intakes) for magnesium intake for European adults have been set at 350 mg/d for men and 300 mg/day for women (8).

Magnesium requirements may be different in CVD patients because of alterations in the cardiovascular system, comorbidities and/or medication use. Mild magnesium depletion is relatively common in users of loop and thiazide diuretics (9), often prescribed to CVD and T2D patients. Hypomagnesemia has been associated with insulin resistance, altered lipid metabolism, impaired endothelial function and kidney function decline (10, 11). A cohort study of 3,380 Chinese CHD patients showed higher risks of all-cause (HR: 1.59, 95% CI: 1.30–1.95) and CVD mortality (HR: 1.71, 95% CI: 1.32–2.22) when comparing higher (≥0.82 mmol/L) to lower serum magnesium concentrations (12). However, only 1–2% of body magnesium is present in the extracellular fluid and serum levels are tightly regulated (13, 14), so serum magnesium may not accurately reflect actual magnesium status (14).

Little is known about magnesium intake and long-term mortality risk in CVD patients. We therefore examined dietary magnesium and risk of CVD, all-cause, and CHD mortality, on top of drug treatment, in Dutch patients aged 60–80 y who had experienced a myocardial infarction (MI).

## Methods

### Design and study population

The Alpha Omega Cohort (AOC; ClinicalTrials.gov Identifier NCT03192410) is an ongoing follow-up of the Alpha Omega Trial (15). During the initial trial phase, MI patients were randomized to either receiving low doses of omega-3 fatty acids or placebo for 40 months, which showed no effect on major CVD events (16). Participants include Dutch males and females aged 60–80 y with a clinically diagnosed MI ≤10 y prior to study enrolment, most of whom received state-of-the-art cardiovascular drug treatment. After the trial, patients have continuously been followed for cause-specific mortality. Patients provided written informed consent and the study was approved by a central ethics committee (Haga Hospital, The Hague, The Netherlands) and by the ethics committees of participating hospitals.

Of 4,837 patients enrolled in the AOC between 2002 and 2006, 472 patients were excluded because of missing or incomplete dietary data (*n* = 453), or implausibly high or low energy intakes (<800 or >8,000 kcal/day for males, <600 or >6,000 kcal/day for females, *n* = 19). One participant was lost to follow-up and censored after 2.9 y. A total of 4,365 patients was included in the analysis (flow-chart of selection of population for analyses in Supplementary Figure 1).

### Dietary assessment

Dietary intake data were collected at baseline by a 203-item food frequency questionnaire (FFQ) developed for the Alpha Omega Cohort. The FFQ is an extended and adapted version of a reproducible and biomarker-validated FFQ (17). Patients were asked to report their habitual intake of foods and beverages consumed during the previous month. The FFQ included questions on the frequency, amount, and type of foods, as well as preparation methods. Trained dietitians checked the questionnaires upon return and obtained additional information by phone on unclear or missing items (15). Daily intakes of foods were linked with the 2006 Dutch Food Composition Database, whereafter energy, macronutrients and micronutrient, including magnesium intake, were calculated (18). Adequate magnesium intakes were calculated based on European magnesium guidelines and set at 350 mg/d for men and 300 mg/day for women (8). Total magnesium intake was categorized into tertiles of energy adjusted magnesium intake (low; <283 mg/d, moderate; 283–322 mg/d and high >322 mg/d). The 2015 Dutch Healthy Diet index (DHD15-index) score was calculated to reflect adherence to Dutch dietary guidelines [DHD15-index; scale from 0 to maximal adherence (0–150)] (19). Dietary supplement use was assessed at baseline by means of a self-administered Lifestyle and Health questionnaire. Information was obtained on types of supplements (including magnesium containing supplements), brand names, frequency of use and daily dosages.

### All-cause mortality and cause-specific mortality

The study focuses on CVD mortality as the primary endpoint, and CHD mortality and all-cause mortality as secondary endpoints. Information on vital status and cause of death was obtained from baseline through 31 December 2018. In the period 2002–2009 (Alpha Omega Trial), an independent Endpoint Adjudication Committee assigned causes of death using information from the national mortality registry [Statistics Netherlands, (CBS)], treating physicians, and close family members, as described previously (15, 16). In the period 2010–2018, research staff assigned causes of death using information from CBS and additional information by treating physicians. Mortality coding was performed according to the International Classification of Diseases, tenth revision (ICD-10) (20), combining primary and secondary causes of death. CVD mortality comprised I20–I25 (ischemic heart disease), I46 (cardiac arrest), R96 (sudden death, undefined), I50 (heart failure), and I60–I69 (stroke). CHD mortality comprised ICD-10 codes I20–I25, I46, and R96. Person-years were calculated from the date of study enrolment to date of death or end of the study (31 December 2018), whichever came first.

### Other measurements

Information on demographics, anthropometrics, lifestyle factors, medical history, and medication use was collected at baseline. Physical examinations were performed by trained research nurses. Body Mass Index (BMI) was calculated as weight (kg) divided by height squared (m^2^). Obesity was classified as BMI ≥30 kg/m^2^. Systolic and diastolic blood pressures were measured twice with an automatic device, in a seated position, after a 10-min rest. Blood lipids and glucose levels were analyzed by standard kits by using an autoanalyzer (Hitachi 912; Roche Diagnostics). Information on chronic disease history, smoking habits (never, former >10 y, former ≤10 y or current), educational level (only elementary, low, intermediate or high) and medication use were collected by a self-administered questionnaire.

The prevalence of diabetes mellitus was defined on basis of a self-reported physician's diagnosis, use of antidiabetic medication, and/or elevated plasma glucose (≥7.0 mmol/L when fasted or ≥11.1 mmol/L when not fasted). Kidney function was assessed on basis the estimated glomerular filtration rate (eGFR) using the CKD-EPI formula, and categorized as impaired (eGFR < 60 ml/min/1.73 m^2^) or unimpaired (eGFR ≥ 60 ml/min/1.73 m^2^). Medication use was checked by research nurses and coded according to the Anatomical Therapeutic Chemical Classification System (21) as follows: C02, C03, C07, C08, and C09 for antihypertensive drugs and C10 for lipid-modifying drugs. Diuretics were coded as C03, with C03A for thiazide diuretics and C03D + CO3E for potassium-sparing diuretics.

Sex-specific categories were used for alcohol intake, since females may have higher and more prolonged blood levels of alcohol compared to males for the same dose of alcohol per kg of body weight (22). Alcohol intake (g/d) was derived from the FFQ and categorized as “no/light drinking” (males: <10 g/d, females: <5 g/d), “moderate drinking” (males: ≥10–30 g/d, females: ≥5–15 g/d) and “heavy drinking” (males: ≥30 g/d, females: ≥15 g/d). Physical activity was assessed by the validated Physical Activity Scale for the Elderly (PASE) (23) and categorized as low physical activity [ <3 Metabolic Equivalent Tasks (METs)], intermediate physical activity [>0 to <5 days per week of moderate or vigorous activity (≥3 METs)], or high physical activity (≥5 days per week of moderate or vigorous activity), based on the Dutch Physical Activity Guidelines (24).

### Statistical analysis

Baseline characteristics in tertiles of energy adjusted magnesium intake (mg/d) are presented as mean ± standard deviations (SDs) for normally distributed variables, medians with IQRs for skewed variables, and counts (*n*) including percentages (%) for categorical or dichotomous variables. Missing data (assuming missing at random) were imputed with the age and sex specific median (for continuous variables) or mode (for categorical variables). Variables that contain imputed values are BMI (*n* = 6), educational level (*n* = 24), physical activity (*n* = 25) and smoking status (*n* = 1). Multicollinearity was assessed by making use of the variance inflation factor (VIF. Multicollinearity was considered as present when VIF was higher than 5 (25). Magnesium intake was adjusted for total energy using the residual method by Willett et al. (26).

Cox proportional hazard models were used to assess the associations of energy adjusted magnesium intake in tertiles, and the risk of CVD mortality, all-cause mortality and CHD mortality. The proportional hazards assumption was checked by log-minus-log plots and was met. Survival time (in y) was defined as the period between the date of inclusion and the date of death, censoring date, or end of follow-up (31 December 2018), whichever occurred first. HRs with 95% CIs were computed in tertiles of energy adjusted magnesium intake, using the lowest tertile as the reference.

HR were adjusted for age and sex (model 1). Model 2 additionally included smoking (4 categories), alcohol intake (3 categories), physical activity (3 categories), obesity (yes/no) and education level (4 categories). Model 3 also included dietary factors, i.e., daily intake of calcium, vitamin D, sodium (only from foods), potassium, heme iron, vitamin C, beta-carotenoids, polyunsaturated fatty acids, saturated fatty acids and total energy, prescribed fiber-rich diet (yes/no) and DHD15-index (total score). Model 4 additionally included blood pressure, kidney function (2 categories) and diabetes mellitus (yes/no), which could be potentially intermediary factors when studying magnesium intake and mortality risk.

Restricted cubic splines (RCS) were used to examine the continuous associations for energy-adjusted magnesium intake and CVD mortality (full model) and to detect a potential threshold or non-linear associations. For the RCS analysis, the reference value was set at the adequate intake for males (350 g/d) and knots were placed at the 10th, 50th, and 90th percentiles. Outliers were winsorized at the 1st and 99th percentile, meaning that outliers outside these percentiles (0–1 percentile and 99–100 percentile) were replaced with the observations closest to them.

Analyses for CVD, all-cause and CHD mortality were repeated in predefined strata by sex, diabetes mellitus (yes vs. no: 883 vs. 3,482 patients), impaired kidney function (yes vs. no: 971 vs. 3,394 patients) and use of diuretics (yes vs. no: 1,050 vs. 3,315 patients), using the full model. For diuretic and non-diuretic users, RCS plots were additionally constructed to obtain further insight in potential effect modification. Since diuretics are commonly prescribed for diabetic patients, associations between dietary magnesium and CVD mortality could be affected by diabetes status. Therefore, a sensitivity analysis excluding patients with diabetes (*n* = 310) from the subgroup of diuretic users (*n* = 1,050) was performed. To get insight in the type of diuretic that could affect the association between magnesium and CVD mortality, two additional sensitivity analyses were performed excluding thiazide users (*n* = 165) and potassium-sparing diuretic users (*n* = 65). In another sensitivity analysis, patients using magnesium containing supplements (*n* = 235) were excluded. Finally, subgroup analyses were performed in strata of total iron intake (low vs. high based on median intake of 10.2 mg/d) and fiber intake (low vs. high based on the median intake of 21.0 g/d), because of possible correlation with magnesium in the diet.

SPSS version 25.0 (SPSS, Inc. Chicago, IL) was used for all analyses. Two-sided *P*-values <0.05 indicated statistical significance.

## Results

Patients were on average 69 ± 5.6 y old and 21% was female. Patients had their last MI on average 4.3 ± 3.2 y ago. The mean magnesium intake was 302 ± 78 mg/day, with 960 out of 3,432 male (28%) and 306 out of 933 (33%) female patients having adequate intakes. Magnesium containing supplements were used by 235 out of 4,365 patients (5.4%). Baseline characteristics in tertiles of energy adjusted dietary magnesium are shown in Table 1. Patients with a higher magnesium intake were more often highly educated, physically active, had a lower prevalence of impaired kidney function and were less often current smokers or categorized as heavy drinkers.

**Table 1:** Baseline characteristics of 4,365 post-MI patients from the Alpha Omega Cohort, by tertiles of energy adjusted magnesium intake^a^.

	**Tertiles of energy adjusted magnesium intake**	
	** <283 mg/d (*n* = 1,453)**	**283–322 mg/d (*n* = 1,459)**	**>322 mg/d (*n* = 1,453)**
Age, y	69.5 (60.5–78.5)	68.9 (59.9–77.9)	68.1 (59.1–77.1)
Females	236 (16)	348 (24)	349 (24)
Dutch ethnicity^b^	1,423 (98)	1,416 (97)	1,425 (98)
BMI, kg/m^b, 3^	27.2 (22.2–32.2)	27.4 (22.4–32.4)	27.2 (23.4–31.4)
Obese	321 (22)	368 (25)	343 (24)
**Educational level** ^**d**^			
Only elementary	347 (24)	281 (19)	254 (18)
Low	530 (37)	529 (36)	498 (34)
Intermediate	427 (29)	473 (32)	467 (32)
High	141 (10)	168 (12)	226 (16)
**Smoking status** ^**e**^			
Never	158 (11)	266 (18)	298 (21)
Former; quit >10 y ago	208 (14)	272 (19)	287 (20)
Former; quit ≤ 10 y ago	768 (53)	710 (49)	684 (47)
Current	319 (22)	210 (14)	184 (13)
**Physical activity** ^**f**^			
Low	710 (49)	581 (40)	492 (34)
Intermediate	499 (34)	569 (39)	567 (39)
High	240 (17)	300 (21)	382 (26)
**Alcohol consumption** ^**g**^			
No or light drinking	802 (55)	817 (56)	840 (58)
Moderate drinking	391 (27)	414 (28)	407 (28)
Heavy drinking	260 (18)	226 (16)	206 (14)
Time since last MI, y^h^	3.7 (0–8.7)	3.6 (0–8.6)	3.4 (0–8.4)
Diabetes mellitus^i^	264 (18)	318 (22)	301 (21)
Impaired kidney function^j^	377 (26)	322 (22)	272 (19)
**Blood pressure, mmHg** ^**k**^			
Systolic	140.0 (112.0–168.0)	141.5 (111.5–171.5)	140.0 (111.0–169.0)
Diastolic	80.0 (65.0–95.0)	80.0 (66.0–94.0)	79.5 (64.5–94.5)
**Serum lipids, mmol/L**			
LDL cholesterol^l^	2.5 (1.5–3.5)	2.5 (1.5–3.5)	2.5 (1.5–3.5)
HDL cholesterol^m^	1.2 (1.2–1.2)	1.2 (1.2–1.2)	1.3 (1.3–1.3)
**Use of cardiovascular medication**			
Antihypertensive drugs	1,299 (89)	1,321 (90)	1.308 (90)
Statins	1,214 (84)	1,265 (87)	1,268 (87)
Diuretics	367 (25)	341 (23)	342 (24)
**Dietary intake**			
Total energy, kJ/d	1,911 (1,164–2,658)	1,788 (1,159–2,417)	1,919 (1,293–2,545)
Dietary fiber, g/d	17 (10–24)	20 (13–27)	25 (17–33)
Fiber rich diet^n^	69 (5)	158 (11)	219 (15)
Saturated fatty acids, g/d	28 (12–44)	24 (12–36)	23 (11–35)
Polyunsaturated fatty acids, g/d	16 (6–26)	14 (6–22)	13 (5–21)
Sodium, mg/d^o^	1,986 (1,106–2,866)	2,051 (1,197–2,905)	2,353 (1,458–3,248)
Potassium, mg/d	2,770 (1,801–3,739)	3,090 (2,175–4,005)	3,734 (2,723–4,745)
Total iron, mg/d	9 (6–12)	10 (7–13)	12 (6–9)
Heme iron, mg/d	1 (0–2)	1 (0–3)	1 (0–3)
Calcium, mg/d	718 (319–1,117)	814 (421–1,207)	1,015 (520–1,510)
Vitamin D, μg/d	5 (2–8)	4 (2–6)	4 (2–6)
Vitamin C, mg/d	68 (16–120)	84 (26–142)	110 (37–183)
Beta-carotene, μg/d	1,409 (550–2,268)	1,537 (644–2,430)	1,761 (785–2,737)
DHD15-index score^p^	79 (61–97)	80 (62–98)	79 (60–98)

eGFR, estimated glomerular filtration rate; DHD15-index, Dutch Healthy Diet 2015 index; MET, metabolic equivalent task; MI, Myocardial Infarction.

^a^Values are means ± SDs for normally distributed variables, medians (IQRs) for skewed variables, or n (%) for categorical variables, unless otherwise indicated.

^b^Missing data for 76 patients.

^c^Missing data for 6 patients; obesity defined as BMI ≥ 30 kg/m^2^.

^d^Missing data for 24 patients.

^e^Missing data for 1 patient.

^f^Missing data for 25 patients; low activity defined as ≤3 METs, intermediate activity as >3 METs on > 0 to < 5 days per week and high activity as >3 METs on ≥ 5 days per week.

^g^No/light drinking defined as <10 g/d for males and <5 g/d for females, moderate drinking as ≥10–30 g/d for males and ≥5–15 g/d for females and heavy drinking as ≥30 g/d for males and ≥15 g/d for females.

^h^Missing data for 38 patients; MI based on a verified clinical diagnosis <10 y before study enrolment.

^i^Diabetes mellitus based on a self-reported physician's diagnosis, use of antidiabetic medication, and/or elevated plasma glucose (≥7.0 mmol/L when fasted or ≥11.1 mmol/L when not fasted).

^j^Based on CKD-EPI-eGFR <60 ml/min/1.73 m^2^.

^k^Missing data for 6 patients.

^l^Non-fasted, missing data for 309 patients.

^m^Non-fasted, missing data for 111 patients.

^n^Fiber rich diet was self-reported.

^o^Sodium intake only from foods, since discretionary salt use was not assessed by means of the FFQ.

^p^DHD15-index for adherence to the 2015 Dutch dietary guidelines (range 0–100), with higher scores indicating a healthier diet.

During a median follow-up of 12.4 y (48,473 person-y), 2,035 patients died, of which 903 due to CVD causes and 558 due to CHD causes. HRs for CVD mortality, all-cause mortality and CHD mortality by tertiles of energy adjusted dietary magnesium are presented in Table 2, using the lowest tertile as reference (<283 mg/d). Higher magnesium intake (>322 mg/day) was associated with a lower risk of CVD mortality (HR: 0.72; 95% CI: 0.54–0.98) and all-cause mortality (HR: 0.78; 95% CI: 0.64–0.95) in the fully adjusted model. When comparing the moderate to lower tertiles, associations were non-significant for CVD (HR: 0.93; 95% CI: 0.76–1.15) and all-cause mortality (HR: 0.93; 95% CI: 0.81–1.07). Magnesium intake was not significantly associated with CHD mortality (Table 2).

**Table 2:** HRs for magnesium intake in relation to CVD, all-cause and CHD mortality in 4,365 post-MI patients from the Alpha Omega Cohort^a^.

	**Tertiles of energy adjusted magnesium intake**			**Per 100 mg/d**
	** <283 mg/d**	**283–322 mg/d**	**>322 mg/d**	
**CVD mortality**				
Cases	333	307	263	903
Model 1	1.00	0.83 (0.70–1.00)	0.64 (0.53–0.78)	0.67 (0.57–0.79)
Model 2	1.00	0.92 (0.77–1.10)	0.74 (0.60–0.90)	0.75 (0.64–0.89)
Model 3	1.00	0.90 (0.73–1.11)	0.69 (0.51–0.93)	0.58 (0.42–0.80)
Model 4	1.00	0.93 (0.76–1.15)	0.72 (0.54–0.98)	0.62 (0.45–0.86)
**All-cause mortality**				
Cases	750	679	606	2,035
Model 1^b^	1.00	0.86 (0.76–0.96)	0.71 (0.63–0.81)	0.75 (0.67–0.83)
Model 2^c^	1.00	0.96 (0.85–1.08)	0.83 (0.73–0.94)	0.85 (0.76–0.94)
Model 3^d^	1.00	0.91 (0.79–1.04)	0.76 (0.62–0.92)	0.66 (0.54–0.82)
Model 4^e^	1.00	0.93 (0.81–1.07)	0.78 (0.64–0.95)	0.70 (0.57–0.86)
**CHD mortality**				
Cases	201	195	162	558
Model 1	1.00	0.94 (0.75–1.17)	0.69 (0.54–0.89)	0.70 (0.57–0.86)
Model 2	1.00	1.04 (0.83–1.31)	0.80 (0.62–1.04)	0.80 (0.64–0.98)
Model 3	1.00	1.03 (0.79–1.35)	0.78 (0.54–1.15)	0.62 (0.41–0.93)
Model 4	1.00	1.08 (0.83–1.41)	0.84 (0.58–1.24)	0.67 (0.45–1.01)

CVD, cardiovascular disease; CHD, coronary heart disease; MI, myocardial infarction.

^a^Values are HRs (95% CIs) obtained from Cox proportional hazards models, using the lowest tertile as reference.

^b^Adjusted for age and sex.

^c^As model 1, plus smoking, alcohol intake, physical activity, obesity and education level.

^d^As model 2, plus intake of total energy, calcium, vitamin D, sodium (only from foods), potassium, heme iron, vitamin C, beta-carotenoids, polyunsaturated fatty acids, saturated fatty acids, fiber-rich diet and DHD15-Index.

^e^As model 3, plus systolic blood pressure, kidney function and diabetes mellitus.

**Figure 1:**
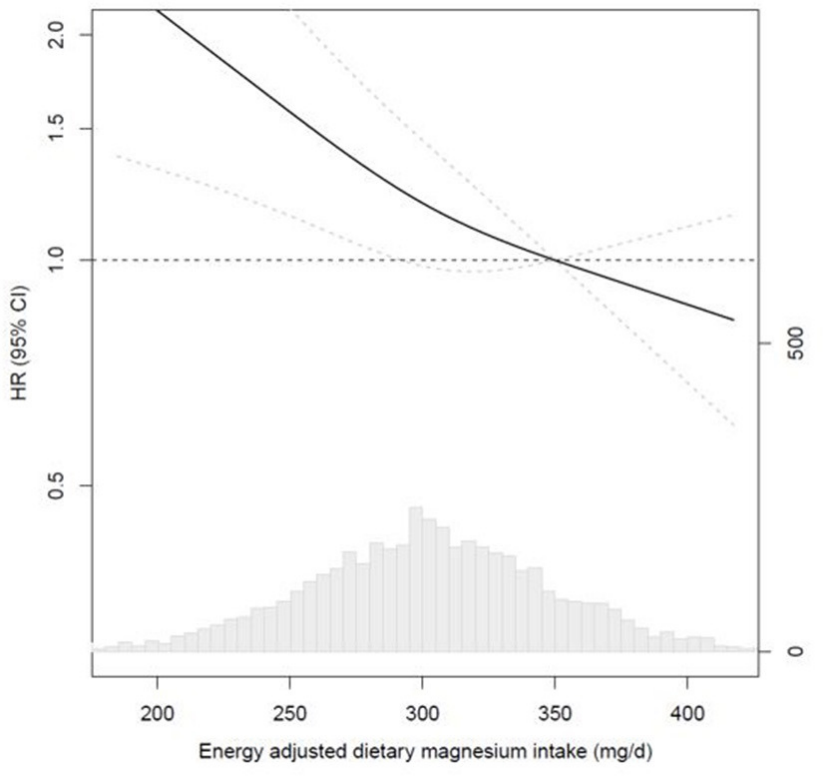
Multivariable-adjusted restricted cubic spline analyses for the continuous association of energy-adjusted magnesium intake with CVD mortality in 4,365 post-MI patients from the Alpha Omega Cohort. HRs for CVD mortality (left y-axis) are presented as a bold line for every level of magnesium intake; the reference (HR of 1.0) was set at 350 mg/d, which is the Adequate Intake for men; HRs were fully adjusted (see Table 3, model 4); knots were placed at the 10th, 50th, and 90th percentile; dotted lines indicate 95% Cis; the bottom graph shows the distribution of magnesium intakes in the Alpha Omega Cohort, with frequencies on the right y-axis. CVD, cardiovascular disease; MI, myocardial infarction.

Figure 1 shows the results from the RCS analyses for energy-adjusted magnesium intake and CVD mortality, using the fully adjusted model and the adequate intake as the reference. Tests for a non-linear association were not statistically significant (*p* = 0.27), indicating a linear association. Highest CVD mortality risks were observed for magnesium intakes below the median intake. Protective risk estimates for CVD mortality were shown for magnesium intakes above the adequate intake.

When analyzing continuously using model 4, dietary magnesium (per 100 mg/d) was associated with CVD mortality (HR: 0.62; 95% CI: 045–0.86) and all-cause mortality (HR: 0.70; 95% CI: 0.57–0.86; Table 2). Borderline significant results were shown for the association between dietary magnesium (per 100 mg/d) and CHD mortality (HR: 0.67; 95% CI: 0.45–1.01; Table 2).

### Subgroup and sensitivity analyses

Subgroup analyses for dietary magnesium and CVD mortality are presented in Table 3. Protective associations were found both in men and women, comparable to the total cohort. Stronger inverse associations were found in patients using diuretics (HR: 0.55 for upper vs. lower tertile; 95% CI: 0.34–0.89), compared to non-diuretic users (HR: 0.89; 95% CI: 0.61–1.30). The presence of diabetes or impaired kidney function did not essentially modify the associations (Table 3). HRs were roughly similar across strata of total iron intake. For dietary fiber, however, stronger associations were found in patients with a relatively low fiber intake (HR: 0.54; 95% CI: 0.32–0.91). Results of subgroup analyses for dietary magnesium in relation to all-cause and CHD mortality (secondary endpoints) are presented in Supplementary Tables 1, 2. Results in subgroups were roughly similar to those for CVD mortality, expect for smaller subgroups like women or impaired kidney function where findings were inconsistent.

**Table 3:** HRs for energy adjusted magnesium intake in relation to CVD mortality in subgroups of post-MI patients from the Alpha Omega Cohort^a^.

	Tertiles of energy-adjusted magnesium intake*		
	** <283 mg/d**	**283–322 mg/d**	**>322 mg/d**
			
**Sex**
Male (*n* = 3,432/682 cases)	1.00	0.91 (0.72–1.16)	0.73 (0.52–1.01)
Female (*n* = 933)	1.00	0.99 (0.62–1.57)	0.66 (0.32–1.34)
**Diuretics** ^**b**^
Users (*n* = 1,050/364)	1.00	0.81 (0.57–1.14)	0.55 (0.34–0.89)
Non-users (*n* = 3,315/539 cases)	1.00	1.11 (0.85–1.46)	0.89 (0.61–1.30)
**Prevalent diabetes** ^**c**^
Yes (*n* = 883/222 cases)	1.00	1.09 (0.69–1.73)	0.64 (0.33–1.22)
No (*n* = 3,482/681 cases)	1.00	0.91 (0.72–1.15)	0.76 (0.54–1.07)
**Kidney function**
eGFR <60 (*n* = 971/280 cases)	1.00	0.92 (0.62–1.37)	0.75 (0.41–1.37)
eGFR ≥60 (*n* = 3,394/591 cases)	1.00	0.92 (0.71–1.18)	0.71 (0.50–1.01)
**Iron intake** ^**d**^
Low (*n* = 2,182/483 cases)	1.00	0.98 (0.74–1.30)	0.71 (0.45–1.14)
High (*n* = 2,183/420 cases)	1.00	0.92 (0.65–1.29)	0.69 (0.45–1.05)
**Fiber intake** ^**e**^
Low (*n* = 2,183/489 cases)	1.00	0.98 (0.74–1.29)	0.54 (0.32–0.91)
High (*n* = 2,182/414 cases)	1.00	1.05 (0.73–1.52)	0.90 (0.58–1.39)

CVD, cardiovascular disease; eGFR, estimated glomerular filtration rate; MI, myocardial infarction.

*Tertile cut-off values based on total cohort.

^a^Values are HRs (95% CIs) obtained from Cox proportional hazards models, using the lowest tertile as the reference. HRs are adjusted for age, sex, smoking, alcohol intake, physical activity, obesity, education level, dietary factors (see Table 2), systolic blood pressure, kidney function and diabetes mellitus (if not used as stratification factor).

^b^Diuretic use was coded according to the Anatomical Therapeutic Chemical Classification System with code C03.

^c^Diabetes mellitus based on a self-reported physician's diagnosis, use of antidiabetic medication, and/or elevated plasma glucose (≥7.0 mmol/L when fasted or ≥11.1 mmol/L when not fasted).

^d^Stratification based on median iron intake (<10.2 vs. ≥ 10.2 mg/d).

^e^Stratification based on median fiber intake (<21.0 vs. ≥ 21.0 g/d).

Sensitivity analyses were performed in the subgroup of diuretic users (Supplementary Table 3, Supplementary Figure 2). After excluding diabetic patients, the association between dietary magnesium and CVD mortality became stronger (HR: 0.47; 95% CI: 0.26–0.83). After excluding thiazide or potassium-sparing diuretic users, the associations became weaker, with HRs around 0.71 in the upper vs. lower tertiles.

Results for CVD mortality were similar after excluding a small group of patients (5.4%) who used magnesium containing supplements (HR: 0.94 in mid vs. lower tertile; 95% CI: 0.75–1.16 and HR: 0.72 in upper vs. lower tertile; 95% CI: 0.53–0.98) (data not shown in table).

## Discussion

This prospective study in 4,365 Dutch patients with a history of MI showed strong inverse associations of dietary magnesium with CVD mortality and all-cause mortality. Associations with CVD mortality were mainly present in patients using diuretics and in patients with a low dietary fiber intake.

The average magnesium intake in our cohort based on FFQ data was around 300 mg/d, which is relatively low. A difference of 100 mg/d in energy adjusted magnesium intake was related to a 30–40% lower risk of CVD and all-cause mortality in (several subgroups of) our cohort. To the best of our knowledge, there are no previous studies of dietary magnesium and mortality in post-MI patients. A meta-analysis of population-based studies by Bagheri et al. showed inverse associations of dietary magnesium with CVD mortality (pooled relative risk (RR): 0.92 for highest vs. lowest comparison; 95% CI: 0.87–0.97, *n* = 9) and all-cause mortality (RR: 0.91 for highest vs. lowest comparison; 95%CI: 0.87–0.96, *n* = 14) when pooling cohort studies that adjusted for total energy intake (comparable to our study). For CHD mortality, we found a HR of 0.67 per 100 mg/d in post-MI patients, which was borderline significant. A meta-analysis by Zhao et al. (554,581 participants) of population-based studies also showed an inverse association with CHD mortality (pooled RR: 0.92 per 100 mg/d, 95% CI: 0.82–0.98) (4). Based on the totality of evidence, we conclude that magnesium intake could be more strongly related to mortality risk in CVD patients than in the general population.

Diuretics may impact magnesium status. Thiazide and potassium-sparing diuretics inhibit sodium transport in the kidney, which indirectly affects magnesium reabsorption (27). In a population-based, cross-sectional study among 9,820 participants, the use of thiazide diuretics was associated with lower serum magnesium levels and a higher risk of hypomagnesaemia (28). In the Alpha Omega Cohort, dietary magnesium tended to be more strongly associated with a lower CVD mortality risk in in diuretic users than in non-users. The prevalence of diabetes in our study was higher in diuretic users (30%) than in non-users (17%). After excluding diabetic patients from the analysis, the stronger association in diuretic users persisted. Based on sensitivity analyses by type of diuretics, we conclude that both thiazide diuretics and potassium-sparing diuretics could be involved in the relationship between magnesium intake and CVD mortality.

Dietary magnesium may interact with dietary iron, which could have an effect on CVD mortality. In our multivariable models, we could only adjust for heme iron (not for total iron) because of multicollinearity (VIF of total iron 7.4, results not shown). We therefore performed an additional stratified analysis by total iron intake, showing similar HRs for magnesium and CVD in patients with lower and higher iron intake. Based on our results, we conclude that the associations that we report for magnesium are (largely) independent of iron intake. It may be hypothesized that dietary fiber, rather than magnesium, contributed to the inverse associations with CVD mortality. Magnesium and fiber are partly derived from the same food sources like fruits and vegetables, cereals and legumes (29), and intakes were highly correlated in our study (VIF-5.5, results not shown). Dietary fiber may influence the absorption of minerals, including magnesium, although this is not yet fully understood (30). Protective associations of dietary fiber against CVD mortality have been found in population-based studies, as summarized in a meta-analysis of 15 cohort studies (RR: 0.91, 95% CI: 0.88–0.94 per 10 g/d increase in dietary fiber) (31). It should be noted, however, that most studies in the fiber-CVD meta-analysis (31) did not correct for magnesium and it is therefore unclear whether cardioprotective associations are attributable to dietary fiber or magnesium. In our present study of the Alpha Omega Cohort, we adjusted for prescribed fiber-rich diets in the multivariable models. Furthermore, we conducted subgroup and sensitivity analyses, in which we showed inverse associations for magnesium both in patients with low and high fiber intake and in (magnesium depleted) diuretic users. Based on the totality of findings, we think that magnesium intake independently of fiber could have contributed to the lower risk of CVD mortality in the Alpha Omega Cohort.

Our study had some limitations. Because of the observational design of the study, we should be aware of confounding. We carefully adjusted for a large number of dietary and lifestyle factors, including an overall healthy diet score. Residual confounding from unknown or imprecisely measured confounders, however, cannot fully be excluded. We used a detailed 203-item FFQ for estimating magnesium intake, but dietary assessment methods based on self-report have several shortcomings. We only assessed magnesium intake at baseline, and not during follow-up. This could have led to misclassification of patients for their true magnesium intake, and attenuation of the associations with mortality in our study. Another limitation is the homogenous study population. Patients in the Alpha Omega Cohort were predominantly male, older, post-MI patients from the Netherlands. Results can therefore not be extrapolated to female, younger or healthy populations. Furthermore, we did not analyze blood concentrations of magnesium. However, magnesium concentrations are tightly regulated and only 1–2% of the total body magnesium is circulating (14).

To conclude, we observed a strong, linear inverse association of dietary magnesium with CVD and all-cause mortality risk after MI, which was most pronounced in patients who used diuretics. Our findings emphasize the importance of an adequate magnesium intake in CVD patients, on top of cardiovascular drug treatment.

## Data availability statement

The raw data supporting the conclusions of this article will be made available by the authors, without undue reservation.

## Ethics statement

The studies involving human participants were reviewed and approved by Central Ethics Committee (Haga Hospital) and by an Ethics Committee in each participating hospital. The patients/participants provided their written informed consent to participate in this study.

## Author contributions

IE conducted the research, performed data-analysis, interpreted the data, wrote the final manuscript, and had primary responsibility for final content. EC performed data-analysis, interpreted the data, and critically reviewed the manuscript. IK performed data-analysis, drafted the first version of the manuscript, and critically reviewed the manuscript. MB supervised the data-analysis and critically reviewed the manuscript. RW critically reviewed the manuscript. JG conceived and designed the study, performed data acquisition, critically reviewed the manuscript, and had primary responsibility for final content. All authors read and approved the final manuscript.

## Funding

The Alpha Omega Cohort was funded by the Netherlands Heart Foundation (grant 200T401) and the National Institutes of Health (NHLBI/ODS grant R01 HL 076200). The research presented in this paper was supported by a grant from Regio Deal Foodvalley (162135). The funding sources had no role in the study design and conduct, data analysis, or manuscript preparation.

## Conflict of interest

The authors declare that the research was conducted in the absence of any commercial or financial relationships that could be construed as a potential conflict of interest.

## Publisher's note

All claims expressed in this article are solely those of the authors and do not necessarily represent those of their affiliated organizations, or those of the publisher, the editors and the reviewers. Any product that may be evaluated in this article, or claim that may be made by its manufacturer, is not guaranteed or endorsed by the publisher.
