# Supplementary material for: Dietary magnesium and risk of cardiovascular and all-cause mortality after myocardial infarction: A prospective analysis in the Alpha Omega Cohort
Source: Front Cardiovasc Med. 2022 Aug 12;9:936772. doi: 10.3389/fcvm.2022.936772 (PMC9416912; doi:10.3389/fcvm.2022.936772)
Supplement: Supplementary file 1 [file Data_Sheet_1.pdf]

Supplementary Table 1 Baseline characteristics of 4,365 post-MI patients from the Alpha Omega Cohort, by tertiles of magnesium intake[1]

	Energy-adjusted tertiles of dietary magnesium		
	<283 mg/d (*n* = 1,453)	283 – 322 mg/d (*n* = 1,459)	>322 mg/d (*n* = 1,453)
Age, y	69.4 ± 5.6	69.1 ± 5.6	68.5 ± 5.5
Females	236 (16)	348 (24)	349 (24)
Dutch ethnicity[2]	1,423 (98)	1,416 (97)	1,425 (98)
BMI, kg/m^2 [3]	27.6 ± 3.9	27.9 ± 3.8	27.7 ± 3.7
Obese	321 (22)	368 (25)	343 (24)
Educational level[4]			
Only elementary	347 (24)	281 (19)	254 (18)
Low	530 (37)	529 (36)	498 (34)
Intermediate	427 (29)	473 (32)	467 (32)
High	141 (10)	168 (12)	226 (16)
Smoking status[5]			
Never	158 (11)	266 (18)	298 (21)
Former; quit >10y ago	208 (14)	272 (19)	287 (20)
Former; quit≤10y ago	768 (53)	710 (49)	684 (47)
Current	319 (22)	210 (14)	184 (13)
Physical activity[6]			
Low	710 (49)	581 (40)	492 (34)
Intermediate	499 (34)	569 (39)	567 (39)
High	240 (17)	300 (21)	382 (26)
Alcohol consumption[7]			
No or light drinking	802 (55)	817 (56)	840 (58)
Moderate drinking	391 (27)	414 (28)	407 (28)
Heavy drinking	260 (18)	226 (16)	206 (14)
Time since last MI, y[8]	4.4 ± 3.6	4.2 ± 3.0	4.2 ± 3.2
Diabetes mellitus[9]	264 (18)	318 (22)	301 (21)
Impaired kidney function[10]	377 (26)	322 (22)	272 (19)
Blood pressure, mmHg[11]			
Systolic	141.7 ± 21.8	142.4 ± 21.6	141.7 ± 21.2
Diastolic	80.4 ± 11.6	80.3 ± 11.1	80.1 ± 10.7
Serum lipids, mmol/L			
LDL cholesterol[12]	2.6 ± 0.9	2.5 ± 0.8	2.6 ± 0.8
HDL cholesterol[13]	1.3 ± 0.3	1.3 ± 0.3	1.3 ± 0.3
Use of cardiovascular medication			
Antihypertensive drugs	1,299 (89)	1,321 (90)	1.308 (90)
Statins	1,214 (84)	1,265 (87)	1,268 (87)
Diuretics	367 (25)	341 (23)	342 (24)
Dietary intake			
Total energy, kJ/d	1,952 ± 563	1,817 ± 481	1,973 ± 504
Dietary fibre, g/d	17 (10-24)	20 (13-27)	25 (17-33)
Fibre rich diet[14]	69 (5)	158 (11)	219 (15)
Saturated fatty acids, g/d	28 (12-44)	24 (12-36)	23 (11-35)
Polyunsaturated fatty acids, g/d	16 (6-26)	14 (6-22)	13 (5-21)
Sodium, mg/d[15]	1,986 (1,106-2,866)	2,051 (1,197-2,905)	2,353 (1,458-3,248)
Potassium, mg/d	2,770 (1,801-3,739)	3,090 (2,175-4,005)	3,734 (2,723-4,745)
Total iron, mg/d	9 (6-12)	10 (7-13)	12 (6-9)
Heme iron (mg/d)	1 (0-2)	1 (0-3)	1 (0-3)
Calcium, mg/d	718 (319-1,117)	814 (421-1,207)	1,015 (520-1,510)

Vitamin D, µg/d	5 (2-8)	4 (2-6)	4 (2-6)
Vitamin C, mg/d	68 (16-120)	84 (26-142)	110 (37-183)
Beta-carotenoids, µg/d	1,409 (550-2,268)	1,537 (644-2,430)	1,761 (785-2,737)
DHD15-index score[16]	79 (61-97)	80 (62-98)	79 (60-98)

[1] Values are means ± SDs for normally distributed variables, medians (IQRs) for skewed variables, or n (%) for categorical variables, unless otherwise indicated.

eGFR, estimated glomerular filtration rate; DHD15-index, Dutch Healthy Diet 2015 index; MET, metabolic equivalent task; MI, Myocardial Infarction.

[2] Missing data for 76 patients.

[3] Missing data for 6 patients; obesity defined as BMI ≥ 30 kg/m^2.

[4] Missing data for 24 patients.

[5] Missing data for 1 patient.

[6] Missing data for 25 patients; low activity defined as ≤3 METs, intermediate activity as >3 METs on > 0 to < 5 days per week and high activity as >3 METs on ≥ 5 days per week.

[7] No/light drinking defined as <10 g/d for males and <5 g/d for females, moderate drinking as ≥10-30 g/d for males and ≥5-15 g/d for females and heavy drinking as ≥30 g/d for males and ≥15 g/d for females.

[8] Missing data for 38 patients; MI based on a verified clinical diagnosis <10 y before study enrolment.

[9] Diabetes mellitus based on a self-reported physician's diagnosis, use of antidiabetic medication, and/or elevated plasma glucose (≥7.0 mmol/L when fasted or ≥11.1 mmol/L when not fasted).

[10] Based on CDK-EPI-eGFR <60 ml/min/1.73 m^2.

[11] Missing data for 6 patients.

[12] Non-fasted, missing data for 309 patients.

[13] Non-fasted, missing data for 111 patients.

[14] Fibre rich diet was self-reported.

[15] Sodium intake only from foods, since discretionary salt use was not assessed by means of the FFQ.

[16] DHD15-index for adherence to the 2015 Dutch dietary guidelines (range 0-100), with higher scores indicating a more healthy diet.

Supplementary Table 2 HRs for magnesium intake in relation to all-cause, CVD and CHD mortality in 4,365 post-MI patients from the Alpha Omega Cohort.[1]

	Energy-adjusted tertiles of dietary magnesium			Per 100 mg/d
	<283 mg/d	283 – 322 mg/d	>322 mg/d	
All-cause mortality				
Cases	750	679	606	2,035
Model 1[2]	1.00	0.86 (0.76 – 0.96)	0.71 (0.63 - 0.81)	0.75 (0.67 - 0.83)
Model 2[3]	1.00	0.96 (0.85 – 1.08)	0.83 (0.73 - 0.94)	0.85 (0.76 - 0.94)
Model 3[4]	1.00	0.91 (0.79 – 1.04)	0.76 (0.62 – 0.92)	0.66 (0.54 – 0.82)
Model 4[5]	1.00	0.93 (0.81 – 1.07)	0.78 (0.64 - 0.95)	0.70 (0.57 - 0.86)
CVD mortality				
Cases	333	307	263	903
Model 1	1.00	0.83 (0.70 – 1.00)	0.64 (0.53 - 0.78)	0.67 (0.57 - 0.79)
Model 2	1.00	0.92 (0.77 – 1.10)	0.74 (0.60 - 0.90)	0.75 (0.64 - 0.89)
Model 3	1.00	0.90 (0.73 – 1.11)	0.69 (0.51 - 0.93)	0.58 (0.42 - 0.80)
Model 4	1.00	0.93 (0.76 – 1.15)	0.72 (0.54 – 0.98)	0.62 (0.45 – 0.86)
CHD mortality				
Cases	201	195	162	558
Model 1	1.00	0.94 (0.75 – 1.17)	0.69 (0.54 - 0.89)	0.70 (0.57 - 0.86)
Model 2	1.00	1.04 (0.83 – 1.31)	0.80 (0.62 – 1.04)	0.80 (0.64 – 0.98)
Model 3	1.00	1.03 (0.79 – 1.34)	0.78 (0.54 – 1.15)	0.62 (0.41 – 0.93)
Model 4	1.00	1.08 (0.83 – 1.41)	0.84 (0.58 – 1.24)	0.67 (0.45 – 1.01)

CVD, cardiovascular disease; CHD, coronary heart disease; MI, myocardial infarction.

[1] Values are HRs (95% CIs) obtained from Cox proportional hazards models, using the lowest tertile as reference.

[2] Adjusted for age and sex.

[3] As model 1, plus smoking, alcohol intake, physical activity, obesity and education level.

[4] As model 2, plus intake of total energy, calcium, vitamin D, sodium (only from foods), potassium, heme iron, vitamin C, beta-carotenoids, polyunsaturated fatty acids, saturated fatty acids, and fibre-rich diet.

[5] As model 3, plus systolic blood pressure, kidney function and diabetes mellitus.

Supplementary Table 3 HRs for magnesium intake in relation to CVD mortality in subgroups of post-MI patients from the Alpha Omega Cohort.[1]

	Energy-adjusted tertiles of dietary magnesium		
	<283 mg/d	283 – 322 mg/d	>322 mg/d
Total cohort	1.00	0.93 (0.76 – 1.15)	0.72 (0.54 – 0.98)
Sex			
Male (n=3,432)	1.00	0.91 (0.72 – 1.16)	0.73 (0.52 – 1.01)
Female (n=933)	1.00	0.99 (0.62 – 1.57)	0.66 (0.32 – 1.34)
Diuretics[2]			
Users (n=1,0503)	1.00	0.81 (0.57 – 1.14)	0.55 (0.34 – 0.89)
Non-users (n=3,315)	1.00	1.11 (0.85 – 1.46)	0.89 (0.61 – 1.30)
Prevalent diabetes[3]			
Yes (n=883)	1.00	1.09 (0.69 – 1.73)	0.64 (0.33 – 1.22)
No (n=3,482)	1.00	0.91 (0.72 – 1.15)	0.76 (0.54 – 1.07)
Kidney function			
eGFR <60 (n=971)	1.00	0.92 (0.62 – 1.37)	0.75 (0.41 – 1.37)
eGFR ≥60 (n=3,394)	1.00	0.92 (0.71 – 1.18)	0.71 (0.50 – 1.01)
Iron intake[4]			
Low (n=2,182)	1.00	0.98 (0.74 – 1.30)	0.71 (0.45 – 1.14)
High (n=2,183)	1.00	0.92 (0.65 – 1.29)	0.69 (0.45 – 1.05)
Fibre intake[5]			
Low (n=2,183)	1.00	0.98 (0.74 – 1.29)	0.54 (0.32 – 0.91)
High (n=2,182)	1.00	1.05 (0.73 – 1.52)	0.90 (0.58 – 1.39)

CVD, cardiovascular disease; eGFR, estimated glomerular filtration rate; MI, myocardial infarction.

[1] Values are HRs (95% CIs) obtained from Cox proportional hazards models, using the lowest tertile as the reference. HRs are adjusted for age, sex, smoking, alcohol intake, physical activity, obesity, education level, dietary factors (see Table 2), systolic blood pressure, kidney function and diabetes mellitus (if not used as stratification factor).

[2] Diuretic use was coded according to the Anatomical Therapeutic Chemical Classification System with code C03.

[3] Diabetes mellitus based on a self-reported physician's diagnosis, use of antidiabetic medication, and/or elevated plasma glucose (≥7.0 mmol/L when fasted or ≥11.1 mmol/L when not fasted).

[4] Stratification based on median iron intake (<10.2 vs ≥ 10.2 mg/d).

[5] Stratification based on median fibre intake (<21.0 vs ≥ 21.0 g/d).

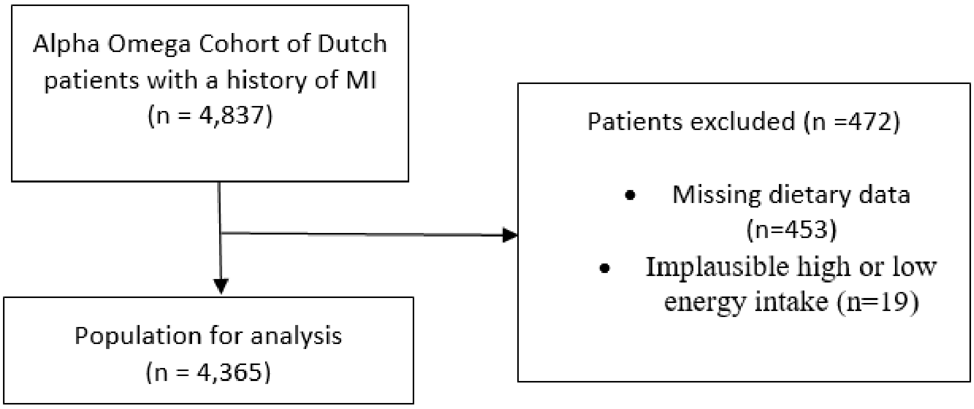

Supplementary figure 1 Flow diagram for selecting the population for analysis from the Alpha Omega Cohort.

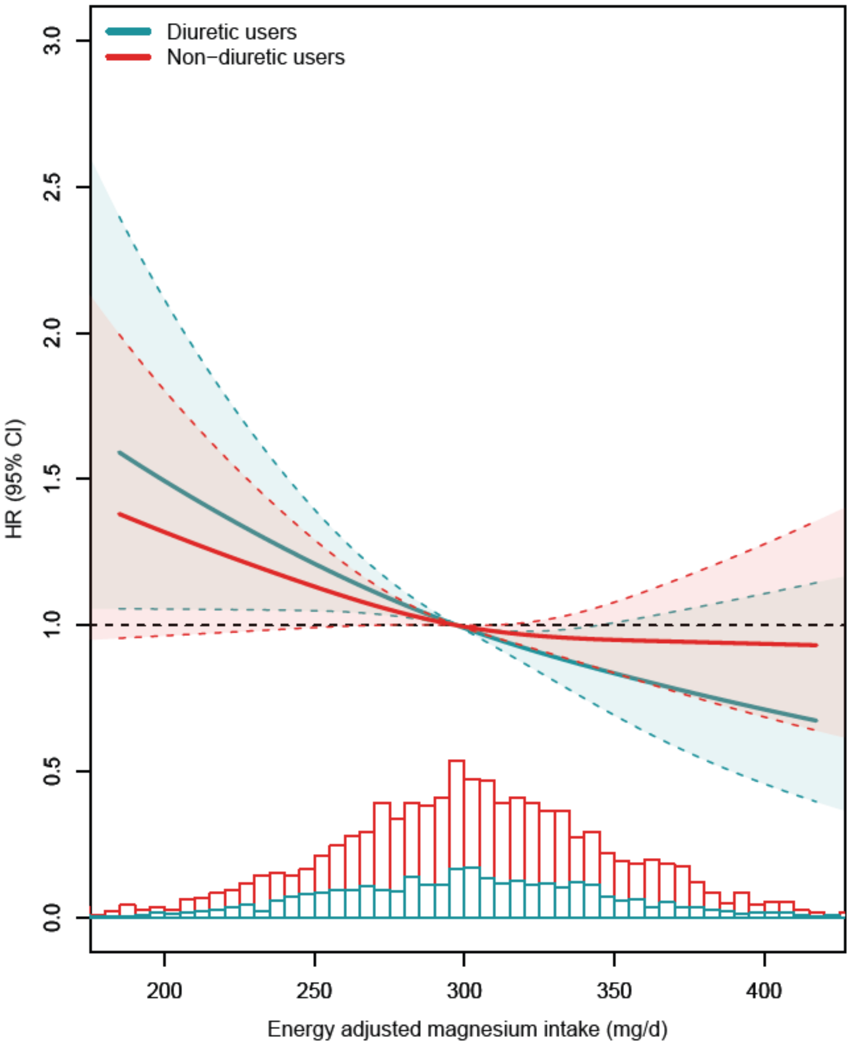

Supplementary figure 2 Multivariable-adjusted restricted cubic spline analyses for the continuous association of energy-adjusted magnesium intake with CVD mortality in diuretic users (n=1,050) and non-diuretic users (n=3,315) of the Alpha Omega Cohort.
